# Intraoperative Application of the PRAETORIAN Score to Predict Optimal Subcutaneous Implantable Cardioverter‐Defibrillator Placement

**DOI:** 10.1002/joa3.70496

**Published:** 2026-10-11

**Authors:** Ken Yamazaki, Shingo Sasaki, Hiroaki Yokoyama, Yuji Ishida, Shogo Hamaura, Yuichi Toyama, Masaomi Kimura, Hirofumi Tomita

**Affiliations:** ^1^ Department of Cardiology and Nephrology Hirosaki University Graduate School of Medicine Hirosaki Japan; ^2^ Department of Advanced Management of Cardiac Arrhythmias Hirosaki University Graduate School of Medicine Hirosaki Japan; ^3^ Department of Cardiac Remote Management System Hirosaki University Graduate School of Medicine Hirosaki Japan; ^4^ Department of Advanced Therapeutics for Cardiovascular Diseases Hirosaki University Graduate School of Medicine Hirosaki Japan

**Keywords:** body mass index, intraoperative risk assessment, PRAETORIAN score, S‐ICD

## Abstract

**Background:**

The PRAETORIAN score (PS) estimates the risk of defibrillation failure after subcutaneous implantable cardioverter‐defibrillator (S‐ICD) implantation based on postoperative chest radiography in the standing position. However, the usefulness of assessing the PS in the supine position intraoperatively has not yet been established.

**Methods:**

We evaluated conventional (standing) and intraoperative supine PS in 124 consecutive Japanese patients undergoing S‐ICD implantation. Changes between conventional and supine PS values were analyzed in two ways: (1) the presence of any difference in the absolute PS values regardless of the risk category, and (2) changes in clinically relevant risk categories for conversion failure (low, intermediate, or high risk). Predictors of discordance between the two assessments were also investigated.

**Results:**

The median conventional PS was 30; 116 and 8 patients were classified as having low and intermediate risk of conversion failure, respectively. Numerical differences between the conventional and supine PS values were observed in 13 patients. However, clinically meaningful risk reclassification occurred in only two patients, suggesting that intraoperative supine PS assessment may underestimate PRAETORIAN score–based risk classification in a small subset of patients. Among patients with numerical PS changes, supine PS was significantly lower than conventional PS. An elevated BMI was strongly associated with PS discordance. Exploratory receiver operating characteristic curve analysis identified a BMI cutoff of 25.6 kg/m^2^ for discriminating patients with numerical PS discordance.

**Conclusions:**

Intraoperative supine PS assessment shows excellent concordance with conventional standing PS and may provide a practical tool for confirming appropriate system positioning in most Japanese patients.

## Graphical abstract

Intraoperative supine PRAETORIAN score assessment showed excellent concordance with conventional standing assessment in Japanese patients undergoing S‐ICD implantation. Numerical score changes were associated with elevated BMI, suggesting that careful interpretation is warranted in these patients.

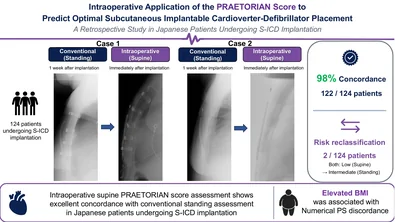

## 1 Introduction

Subcutaneous implantable cardioverter‐defibrillator (S‐ICD) is an established alternative to the conventional transvenous implantable cardioverter‐defibrillator (TV‐ICD), particularly in young patients and those with limited vascular access, owing to its non‐transvenous design. The safety and effectiveness of S‐ICD are comparable to those of TV‐ICD in preventing sudden cardiac death [1, 2, 3, 4]. The clinical utility of S‐ICD therapy has been investigated across various underlying heart diseases, including hypertrophic and dilated cardiomyopathy [5, 6]. In addition, implantable cardioverter‐defibrillator (ICD) therapy improves long‐term survival in patients at risk of sudden cardiac death, and prognostic factors associated with clinical outcomes have been reported in primary prevention populations [7]. Because appropriate system positioning directly influences defibrillation efficacy, optimal intraoperative placement of an S‐ICD is essential to achieve reliable protection against life‐threatening ventricular tachyarrhythmias.

The PRAETORIAN score (PS), introduced in 2019, is a radiographic tool designed to assess the risk of defibrillation failure after S‐ICD implantation, based on chest radiography in the standing position [8]. We previously evaluated the usefulness of the PS in Japanese patients with S‐ICD and found that the majority of patients were classified with a low risk of conversion failure [9]. However, PS calculation requires a standing chest radiograph, which limits its applicability for intraoperative assessment.

This study aimed to determine whether the PS can be reliably applied in the supine position for the intraoperative assessment of Japanese patients undergoing S‐ICD implantation. Accordingly, we evaluated the PS in the supine position immediately after S‐ICD implantation and compared these findings with those of the conventional standing PS assessment.

## 2 Materials and Methods

### 2.1 Study Population

This study included 124 consecutive patients who underwent S‐ICD implantation at Hirosaki University Hospital between February 2016 and November 2021. In this population, S‐ICD was selected as an alternative to TV‐ICD in accordance with the Japanese Heart Rhythm Society guidelines [10] for patients with an ICD indication who did not require pacing therapy. S‐ICD was preferentially selected for patients with difficult venous access, those who had undergone TV‐ICD extraction due to device infection, and young patients expected to require long‐term ICD therapy. The study protocol was approved by the Ethics Committee of Hirosaki University Graduate School of Medicine (IRB No. 2022–030).

### 2.2 S‐ICD Implantation Procedure

The S‐ICD implantation procedure was performed according to the institutional protocol, as previously described [9]. Briefly, body surface marking was performed, and the procedure was performed under conscious sedation with local anesthesia. The pulse generator was placed in the intermuscular space between the serratus anterior and latissimus dorsi muscles, and the subcutaneous electrode was positioned parallel to the left sternal border using the two‐ or three‐incision technique. The device position was evaluated fluoroscopically, and the intraoperative PS in the supine position was assessed during the procedure. Defibrillation testing was performed unless contraindicated.

### 2.3 PRAETORIAN Score Analysis

Postoperative posteroanterior and lateral chest radiographs were obtained in the supine position immediately after implantation, and in the standing position 1 week after surgery. PS was calculated based on three factors known to affect the S‐ICD defibrillation efficacy: (1) adipose tissue width beneath the coil electrode, (2) anteroposterior position of the generator, and (3) adipose tissue width beneath the generator [11].

When the total score derived from these three components was ≥ 90 and body mass index (BMI) was ≤ 25 kg/m^2^, 40 points were subtracted from the total score, reflecting the reduced impact of adipose tissue in patients with a low BMI, as described in the original PS methodology. The PS ranged from 30 to 900 and stratified the risk of conversion failure into three risk categories: low (< 90), intermediate (90–149), and high (≥ 150) [8].

### 2.4 Definition of Numerical PS Change and Risk Reclassification

The PS calculated using supine chest radiography obtained immediately after implantation was defined as intraoperative supine PS (supine PS), whereas the score assessed using standing chest radiography obtained 1 week after surgery was defined as conventional PS.

Changes between supine and conventional PS were assessed in two ways: (1) any difference in the absolute PS values regardless of the risk category, and (2) clinically relevant risk reclassification based on changes in the risk category for conversion failure (low vs. intermediate or high risk) (Figure 1).

**FIGURE 1:**
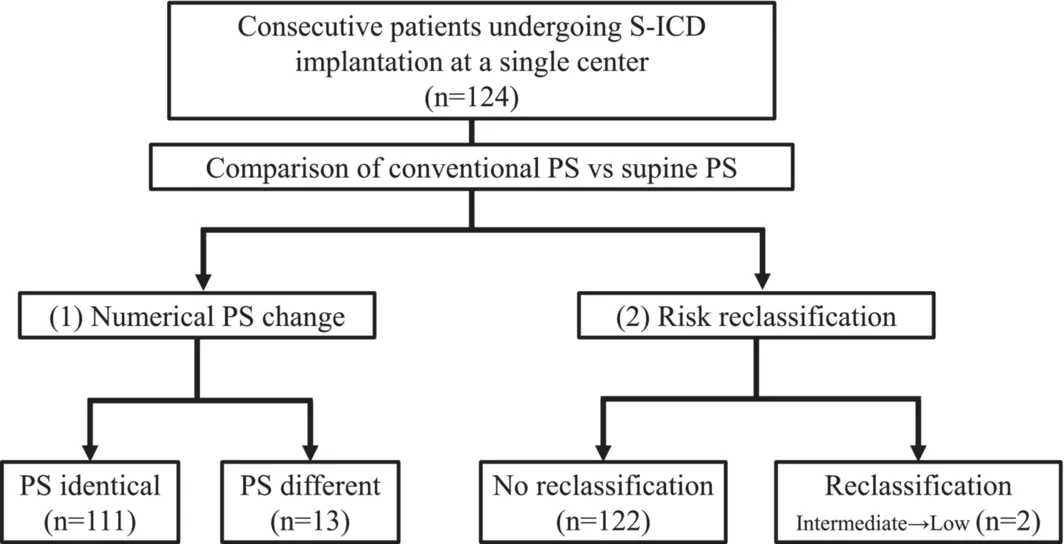
Definition of numerical PRAETORIAN score (PS) change and risk reclassification. Numerical PS change is defined as the absolute difference in PS values between the conventional (standing) and intraoperative supine PS, regardless of the risk category. Risk reclassification is defined as a change in clinically relevant risk categories for conversion failure (low vs. intermediate or high risk) between the two assessments.

Clinical and procedural characteristics were compared based on the presence or absence of numerical PS changes, and the factors associated with PS discordance were explored.

### 2.5 Statistical Analysis

Baseline characteristics are presented as medians (interquartile ranges [IQR]) for continuous variables and numbers (percentages) for categorical variables. Continuous variables were compared using the Mann–Whitney *U* test, whereas paired continuous variables were compared using the Wilcoxon signed‐rank test. Categorical variables were compared using Fisher's exact test.

Factors associated with numerical PS changes were evaluated using multivariable logistic regression, adjusting for age and BMI, and the results were expressed as odds ratios (ORs) with 95% confidence intervals (CIs). Receiver operating characteristic (ROC) curve analysis was performed using R software (version 4.5.3; R Foundation for Statistical Computing, Vienna, Austria) to assess the association between BMI and numerical PS changes. All other statistical analyses were conducted using JMP version 19 (SAS Institute Inc., Cary, NC, USA).

## 3 Results

### 3.1 Study Population and S‐ICD Implantation Procedure

The baseline characteristics of the study population are summarized in Table 1. The median age of the patients was 59 (48–68) years, and 99 patients (80%) were men. The underlying heart diseases included ischemic heart disease with prior myocardial infarction in 45 patients (36%), hypertrophic cardiomyopathy in 17 (14%), Brugada syndrome in 16 (13%), idiopathic ventricular fibrillation in 12 (10%), dilated cardiomyopathy in nine (7%), non‐ischemic cardiomyopathy in nine (7%), cardiac sarcoidosis in three (2%), arrhythmogenic right ventricular cardiomyopathy in three (2%), idiopathic ventricular tachycardia in two (2%), drug‐induced cardiomyopathy in two (2%), valvular heart disease in three (2%), and other conditions in three (3%) patients. The median BMI was 24.0 (21.0–27.0) kg/m^2^, and the median left ventricular ejection fraction was 47.5% (34.2%–62.6%). Forty‐nine patients underwent S‐ICD implantation for the primary prevention of sudden cardiac death. The first 22 patients underwent implantation using the three‐incision technique, whereas the remaining 102 patients (82%) underwent implantation using the two‐incision technique. Defibrillation testing was not performed in 23 patients because of contraindications, including intracardiac thrombus, severe left ventricular systolic dysfunction, acute cerebral infarction, or non‐inducibility of sustained ventricular arrhythmia.

**TABLE 1:** Baseline characteristics of the study patients.

Clinical characteristics	*N* = 124
Age, years	59 (48–68)
Male, *n* (%)	99 (80)
Height, cm	166 (160–171)
Body weight, kg	65 (58–75)
BMI, kg/m^2^	24.0 (21.0–27.0)
LVEF, %	47.5 (34.2–62.6)
Sinus rhythm, *n* (%)	111 (90)
Primary prevention indication, *n* (%)	49 (40)
Shock impedance, *Ω*	64 (57–75)
Time to therapy, sec	13.4 (12.4–14.9)
DT performed, *n* (%)	101 (81)
Successful DT, *n* (%)	101 (100)
Underlying heart diseases, *n* (%)	
Ischemic heart disease	45 (36)
Hypertrophic cardiomyopathy	17 (14)
Brugada syndrome	16 (13)
Idiopathic ventricular fibrillation	12 (10)
Dilated cardiomyopathy	9 (7)
Non‐ischemic cardiomyopathy	9 (7)
Cardiac sarcoidosis	3 (2)
Arrhythmogenic right ventricular cardiomyopathy	3 (2)
Idiopathic ventricular tachycardia	2 (2)
Drug‐induced cardiomyopathy	2 (2)
Valvular heart disease	3 (2)
Others	3 (2)

*Note:* Data are shown as median (IQR) or *n* (%). Non‐ischemic cardiomyopathy excluding dilated cardiomyopathy.

Abbreviations: BMI, body mass index; DT, defibrillation test; LVEF, left ventricular ejection fraction.

### 3.2 Conventional and Supine PRAETORIAN Score Risk Stratification

Based on conventional standing PS, 116 patients (94%) were classified as low risk of conversion failure, whereas eight patients (6%) were classified as intermediate risk. According to supine PS, 118 patients (95%) were classified as low risk and six (5%) as intermediate risk. Risk stratification based on conventional and supine PS was concordant in 122 patients (98%). Risk reclassification occurred in only two patients (2%), both of whom were classified as intermediate risk according to conventional PS but as low risk according to supine PS, resulting in potential underestimation of PRAETORIAN score–based risk classification (Table 2 and Figure 2). Although numerical differences between the two assessments were observed, most patients remained in the same risk category, indicating that these differences were not clinically meaningful in most cases (Table 2). In both patients with risk reclassification (corresponding to patients 5 and 6 in Table S1), the discrepancy was attributable to changes in Step 1 of the PS, reflecting the increased substernal fat width observed on standing chest radiography obtained 1 week after surgery compared with supine chest radiography obtained immediately after surgery (Figure S1).

**TABLE 2:** Comparison of supine and conventional PRAETORIAN scores and step‐wise components.

	Supine PRAETORIAN score				
		Low	Intermediate	High	
Conventional PRAETORIAN score	Low	116	—	—	
	Intermediate	2	6	—	
	High	—	—	—	
		Step 1			
		≤ 1 Coil	1 < Coil < 2	2 < Coil < 3	> 3 Coil
	≤ 1 Coil	105	—	—	—
	1 < Coil < 2	4	7	—	—
	2 < Coil < 3	2	—	6	—
	> 3 Coil	—	—	—	—
		Step 2			
		Posterior	Anterior	> ^1^/_2_ Anterior	
	Posterior	122	—	—	
	Anterior	2	—	—	
	> ^1^/_2_ Anterior	—	—	—	
		Step 3			
		< 1 Generator	≥ 1 Generator		
	< 1 Generator	117	2		
	≥ 1 Generator	3	2		

*Note:* Cases with changes are shown with a gray background.

**FIGURE 2:**
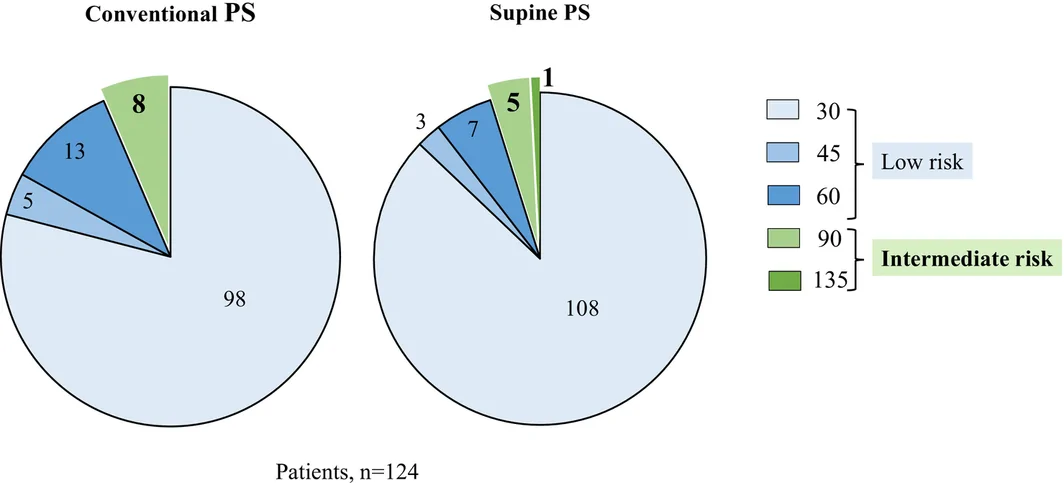
Comparison of conventional and supine PRAETORIAN scores (PSs). Comparison of conventional standing and intraoperative supine PS in the study population. Risk stratification for conversion failure was concordant between the two assessments in 122 patients (98%), indicating high agreement in risk classification.

### 3.3 Numerical Changes in the PRAETORIAN Score Values

Numerical differences between the supine and conventional PS were observed in 13 patients (10%). The individual BMI, supine PS, and conventional PS of these patients are listed in Table S1. No numerical difference was observed in the remaining 111 patients (90%). In the patients with numerical PS changes, supine PS was significantly lower than conventional PS [30 (30–30) vs. 60 (45–75), *p* < 0.05] (Figure 3).

**FIGURE 3:**
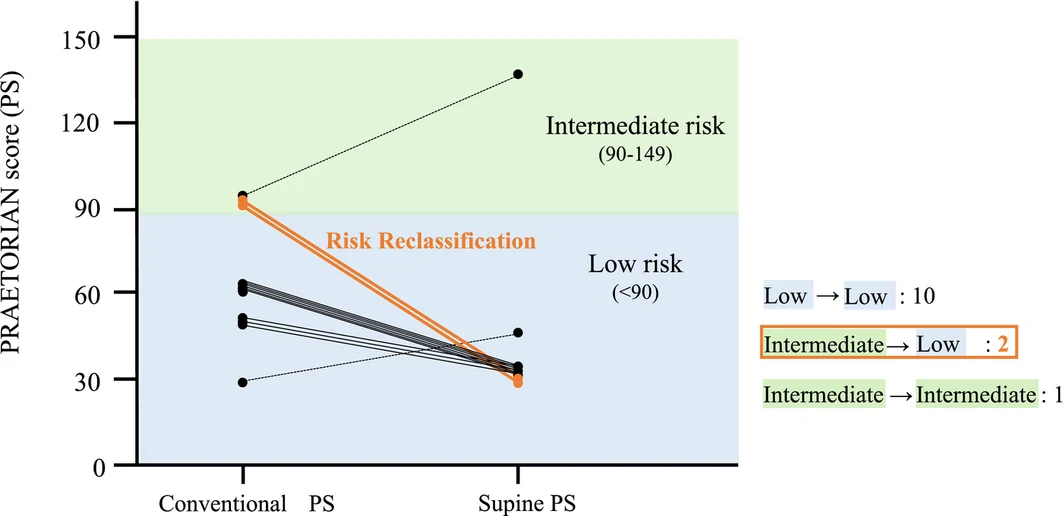
PRAETORIAN score (PS) changes in patients with numerical differences. Comparison of conventional and supine PS in patients with numerical score changes (numerical PS change group, *n* = 13). The supine PS was significantly lower than the conventional score. PRAETORIAN score–based risk classification remained unchanged in 11 patients, whereas two patients were reclassified from intermediate to low risk.

### 3.4 Clinical Characteristics Associated With Numerical PRAETORIAN Score Change

The clinical characteristics were compared between patients with and without numerical changes in PS (Table 3). Age, sex, height, left ventricular ejection fraction, underlying heart disease, or number of incisions did not differ significantly between the two groups. In contrast, BMI was significantly higher in patients with numerical PS changes than in those without changes [29.4 (25.8–31.5) vs. 23.5 (20.8–26.1) kg/m^2^, *p* < 0.05]. In multivariable logistic regression adjusted for age, BMI was independently associated with numerical PS change (OR per 1 kg/m^2^ increase, 1.29; 95% CI, 1.13–1.50; *p* < 0.05) (Table 4). Sex was not included in the multivariable model because all patients in the numerical PS‐change group were men. In ROC curve analysis, BMI emerged as a strong discriminator of numerical PS changes, with an area under the curve of 0.83 (95% CI, 0.74–0.93). A BMI of 25.6 kg/m^2^ was identified as the optimal cutoff for predicting numerical PS changes, yielding a sensitivity of 85% (95% CI, 0.55–0.98) and specificity of 72% (95% CI, 0.63–0.80) (Figure 4).

**TABLE 3:** Comparison of the characteristics between the Numerical PS Change group and the No Numerical PS Change group.

Variables	Numerical PS change group (*n* = 13)	No numerical PS change group (*n* = 111)	*p*
Age, years	59 (48–66)	59 (48–68)	0.91
Male, *n* (%)	13 (100)	86 (77)	0.07
Height, cm	166 (163–174)	165 (160–171)	0.30
BMI, kg/m^2^	29.4 (25.8–31.5)	23.5 (20.8–26.1)	< 0.05
LVEF, %	59 (34–66)	47 (33–62)	0.54
Shock impedance, *Ω*	72 (65–85)	64 (56–74)	0.08
Time to therapy, sec	15.0 (12.0–16.9)	13.3 (12.5–14.8)	0.68
Successful DT, *n* (%)	9/9 (100)	92/92 (100)	0.26

*Note:* Data are shown as median (IQR) or *n* (%). DT was performed in 9 patients in the Numerical PS Change group and 92 patients in the No Numerical PS Change group.

Abbreviations: BMI, body mass index; DT, defibrillation test; LVEF, left ventricular ejection fraction.

**TABLE 4:** Univariate and multivariate analyses for changes between conventional and supine PRAETORIAN score.

Variables	Univariate analysis			Multivariate analysis		
	OR	95% CI	*p*	OR	95% CI	*p*
Age	1.01	[0.97–1.05]	0.68	1.00	[0.96–1.05]	0.97
Male	—	—	—			
Height	1.05	[0.98–1.13]	0.20			
Body weight	1.12	[1.06–1.19]	< 0.05			
BMI	1.29	[1.13–1.50]	< 0.05	1.29	[1.13–1.50]	< 0.05
LVEF	1.01	[0.98–1.05]	0.56			
Shock impedance	1.04	[0.99–1.10]	0.10			
Time to therapy	1.14	[0.92–1.38]	0.22			

Abbreviations: BMI, indicates body mass index; CI, confidence interval; LVEF, left ventricular ejection fraction; OR, odds ratio.

**FIGURE 4:**
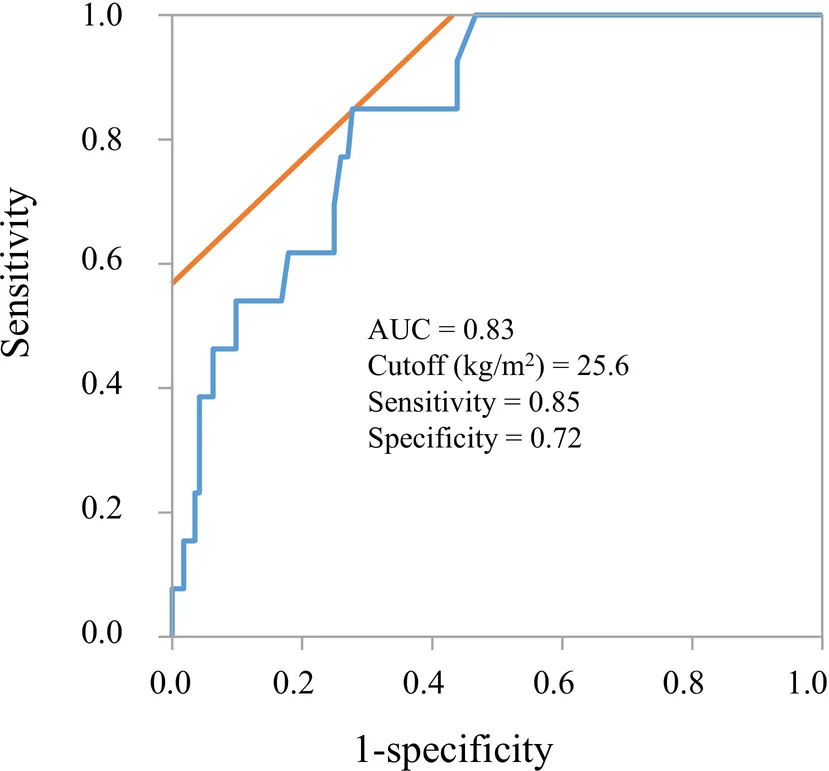
Receiver operating characteristic (ROC) analysis of body mass index (BMI) for predicting numerical PRAETORIAN score (PS) changes. BMI demonstrated good discriminatory ability, with an area under the curve of 0.83 (95% CI, 0.74–0.93). A BMI of 25.6 kg/m^2^ was identified as the optimal cutoff, yielding a sensitivity and specificity of 85% (95% CI, 0.55–0.98) and 72% (95% CI, 0.63–0.80), respectively.

## 4 Discussion

### 4.1 Major Findings

This study demonstrated high concordance in PS‐based risk stratification between supine and conventional standing radiographic assessments in patients undergoing S‐ICD implantation. Although numerical changes in the PS were observed in approximately 10% of the patients, risk reclassification occurred in only 2%, suggesting that these differences were rarely clinically meaningful. These findings support the feasibility of intraoperative supine PS assessment for confirming appropriate system positioning in most patients. However, caution is warranted in patients with an elevated BMI, as clinically relevant risk underestimation may occur. When a low‐risk PS is confirmed on supine chest radiography performed immediately after S‐ICD implantation, it may provide confidence regarding the appropriate system configuration concerning defibrillation efficacy. Our results are consistent with those reported by Gasperetti et al., who demonstrated strong agreement between intraoperative supine PS and conventional standing PS assessments [12].

### 4.2 Supine PRAETORIAN Score Clinical Application

With conventional PS, assessment is performed postoperatively; therefore, neither the physician nor the patient can confirm whether the S‐ICD has been implanted in an optimal position until after the procedure is complete. Consequently, physicians must decide whether patients identified as having an intermediate or high risk of defibrillation failure should undergo reoperation. However, reoperation is associated with several concerns, including the risk of infection, patient discomfort, and increased medical costs. In current clinical practice, reoperation is rarely performed solely based on postoperative PS; instead, careful follow‐up is usually selected. Therefore, patients continue to live with the remaining risk of defibrillation failure.

Intraoperative assessment using supine PS may help to address this issue. Supine PS provides real‐time feedback on the implantation position and can guide system repositioning in patients with high scores, thereby facilitating appropriate system configuration. Among the 124 patients in this study, only two (2%) experienced risk reclassification, both of whom were reclassified from intermediate (conventional PS) to low (supine PS), resulting in potential underestimation of PRAETORIAN score–based risk classification. Notably, all these changes occurred in Step 1 of the PS (Table S1). This finding is consistent with that of Gasperetti et al. [12], who also observed that the risk class did not change, although numerical score changes occurred only in Step 1. Our previous study similarly demonstrated that the subcoil fat depth in Step 1 has a significant impact on PS and the risk of unsuccessful defibrillation, particularly in patients with an elevated BMI [9]. One possible explanation is that Step 1 is particularly susceptible to positional changes because it reflects the amount of adipose tissue beneath the parasternal shock coil. Transition from the supine to the standing position may alter the apparent thickness and distribution of anterior thoracic adipose tissue because of gravitational effects. This may increase the apparent amount of adipose tissue interposed between the sternum and the shock coil, resulting in a higher Step 1 score on standing radiography. In addition, differences in arm positioning, respiratory phase, and imaging projection between intraoperative and postoperative imaging may also contribute to variation in Step 1 assessment. As a potential technical approach, ultrasound‐guided parasternal tunneling may facilitate placement of the shock coil closer to the sternum while minimizing subcoil adipose tissue, thereby improving the accuracy and reproducibility of intraoperative Step 1 assessment. However, this approach requires prospective validation.

In addition to these technical considerations, ICD therapy has also been associated with psychological burden in patients, including anxiety related to returning to work in Japan [13]. Therefore, achieving optimal device positioning at the time of initial implantation may contribute to technical success and improved patient‐centered outcomes.

In this study, the BMI cutoff for predicting a change from supine to conventional PS was 25.6 kg/m^2^. This cutoff should be interpreted as an exploratory finding derived from this cohort rather than as a clinically meaningful or universal threshold, particularly given the ethnic differences in body composition. Notably, the two patients who experienced a change in risk classification had BMI values well above this cutoff. Therefore, we speculate that the optimal BMI cutoff may be slightly higher in the Japanese population, in whom obesity is less prevalent; however, further case accumulation is required. These findings underscore the importance of standardizing parasternal tunneling techniques in patients with an elevated BMI and suggest that intraoperative supine PS evaluation is particularly valuable for achieving optimal S‐ICD implantation in these patients. Because supine PS assessment can be obtained from intraoperative frontal and lateral chest radiographs without significantly prolonging the procedure [14], routine assessment in patients with an elevated BMI appears reasonable and may provide additional clinical benefit. Nevertheless, intraoperative supine PRAETORIAN score assessment should be interpreted with caution in patients with an elevated BMI, as it may underestimate the risk of suboptimal defibrillation and potentially fail to identify patients who may benefit from further optimization of the S‐ICD system. Recent multicenter data from a cohort of more than 1200 patients have shown that the use of an intermuscular generator pocket formation, which is currently recommended and widely adopted in Japan, results in a PS < 90 in over 95% of patients, indicating a low risk of defibrillation failure [14]. In that cohort, BMI was the only independent predictor of an unfavorable score, and all non‐obese patients (< 25 kg/m^2^) exhibited a favorable PS. These findings are consistent with our previous study in Japanese patients and highlight the importance of optimizing parasternal tunneling techniques in individuals with an elevated BMI [9].

In addition to BMI‐based risk stratification, structural assessment using preoperative imaging may further refine patient selection. Kasagawa et al. reported that the amount of adipose tissue adjacent to the device, as quantified by preoperative chest computed tomography (CT), was independently associated with a higher shock impedance [15]. This finding suggests that patients with excessive thoracic adipose tissue may be predisposed to unfavorable shock vectors and reduced defibrillation efficacy, even before implantation. Taken together, patient‐related risk stratification (e.g., BMI and CT‐based adipose assessment) and intraoperative positional optimization using supine PS evaluation should be considered complementary strategies to maximize S‐ICD efficacy, particularly in patients with an elevated BMI. These findings may have practical implications for intraoperative decision‐making, particularly in patients at high risk of suboptimal device positioning. However, because the present study was conducted exclusively in a Japanese population with a relatively low median BMI, caution is warranted when extrapolating these findings to Western populations, in whom obesity and unfavorable PRAETORIAN scores may be more prevalent. Further multicenter studies including more diverse populations are needed to confirm the generalizability of our findings.

### 4.3 Defibrillation Test and PRAETORIAN Score

Defibrillation testing has traditionally been performed to evaluate the safety and efficacy of S‐ICD systems. However, ventricular fibrillation induced during testing is an artificial and transient condition that does not necessarily reflect the complexity of spontaneous clinical arrhythmia. Therefore, successful termination during testing does not guarantee real‐world defibrillation efficacy. Defibrillation efficacy in S‐ICD systems is strongly influenced by the shock impedance and resulting defibrillation threshold (DFT). Adipose tissue increases shock impedance and may elevate the DFT [9, 16]. These findings provide a mechanistic basis for the use of the PS, which incorporates radiographic surrogates of thoracic adipose tissue and device position to estimate the risk of suboptimal defibrillation.

In addition, defibrillation testing may not be feasible in patients with severe heart failure or intracardiac thrombus, further limiting its applicability. In the subanalysis of the PRAETORIAN‐DFT trial [17, 18], a randomized controlled study designed to evaluate the safety of omitting routine defibrillation testing in patients with optimally positioned S‐ICD systems as assessed by the PS, defibrillation testing was successful in almost all patients classified as low risk, suggesting that routine testing may be unnecessary in this population. Based on our findings, if moderate‐ or high‐risk cases can be identified intraoperatively and repositioned to achieve low risk, the PS may ultimately help reduce the need for invasive defibrillation testing. However, the uniformly successful defibrillation testing in our cohort limited the ability to directly evaluate the predictive value of the PS for clinical defibrillation failure. Moreover, because no clinical defibrillation failures occurred during follow‐up, the ability of the supine PS to predict real‐world defibrillation outcomes could not be validated in the present study. Accordingly, the present findings should be interpreted as demonstrating agreement between two imaging‐based assessment methods rather than validation of the predictive ability of the supine PS for clinical defibrillation outcomes.

### 4.4 Study Limitations

This study has several limitations. First, this was a retrospective, single‐center study, which may have limited the generalizability of our findings. However, consecutive patients undergoing S‐ICD implantation during the study period were included, minimizing selection bias. Second, the sample size was relatively small, particularly for patients exhibiting PS discordance or risk reclassification. Furthermore, because only 13 patients exhibited numerical PS changes, the multivariable logistic regression analysis should be considered exploratory and interpreted with caution due to the potential risk of model instability and overfitting. Third, the follow‐up duration may have been insufficient to assess the long‐term defibrillation efficacy. Fourth, no clinical defibrillation failures occurred, and the supine PS could not be validated against clinical defibrillation outcomes. Therefore, its ability to predict actual defibrillation efficacy remains uncertain. Finally, the reproducibility of intraoperative supine radiographic acquisition across different centers was not evaluated, which may have influenced the PS assessment. Prospective multicenter studies with large sample sizes and long follow‐up periods are warranted to further clarify and establish the clinical implications of intraoperative supine PS evaluation.

## 5 Conclusions

In the majority of Japanese patients undergoing S‐ICD implantation, supine PS demonstrated high concordance with conventional standing assessment. Intraoperative supine PS evaluation may provide a practical tool for confirming appropriate system positioning in most Japanese patients. However, careful interpretation is required in patients with an elevated BMI because clinically relevant risk underestimation may occur.

## Funding

This work was supported by Sakurai Memorial Fund for Medical Research of the Hirosaki University Graduate School of Medicine.

## Ethics Statement

The study protocol was approved by the Ethics Committee of Hirosaki University Graduate School of Medicine (IRB No. 2022–030).

## Consent

The requirement for informed consent was waived by the Ethics Committee of Hirosaki University Graduate School of Medicine because of the retrospective nature of the study.

## Conflicts of Interest

Dr. Masaomi Kimura is an associate professor of the Department of Advanced Management of Cardiac Arrhythmias, which is an endowment Department supported by Medtronic Japan Co. Ltd. and Japan Lifeline Co. Ltd. Dr. Shingo Sasaki received a research grant from Boston Scientific Japan Co. Ltd. and is a concurrent associate professor of the Department of Advanced Management of Cardiac Arrhythmias and the Department of Cardiac Remote Management System, which is an endowment Department supported by BIOTRONIK Japan Co. Ltd. Dr. Shogo Hamaura is an instructor of the Department of Advanced Therapeutics for Cardiovascular Diseases, which is an endowment Department supported by Boston Scientific Japan Co. Dr. Hirofumi Tomita received a research grant from Abbott Medical Japan LLC. and is a concurrent professor of the Department of Advanced Management of Cardiac Arrhythmias, the Department of Cardiac Remote Management System, and the Department of Advanced Therapeutics for Cardiovascular Diseases. Other authors have no relevant disclosures.

## Supporting information


**Table S1:** Details of PRAETORIAN score and BMI for 13 cases in the Change group.
**Figure S1:** Representative radiographs of patients with risk reclassification between supine and conventional PRAETORIAN score assessments.

## Data Availability

The data that support the findings of this study are available from the corresponding author upon reasonable request.
