# Supplementary material for: Intraoperative Application of the PRAETORIAN Score to Predict Optimal Subcutaneous Implantable Cardioverter‐Defibrillator Placement
Source: J Arrhythm. 2026 Oct 11;42(5):e70496. doi: 10.1002/joa3.70496 (PMC13655365; doi:10.1002/joa3.70496)
Supplement: Supplementary file 1 [file JOA3-42-e70496-s001.doc]

**Supplementary Material**

**Intraoperative Application of the PRAETORIAN Score to Predict Optimal Subcutaneous Implantable Cardioverter-Defibrillator Placement**

Ken Yamazaki, MD, PhD, Shingo Sasaki, MD, PhD, Hiroaki Yokoyama, MD, PhD, Yuji Ishida, MD, PhD, Shogo Hamaura, MD, Yuichi Toyama, MD, PhD, Masaomi Kimura, MD, PhD, and Hirofumi Tomita, MD, PhD.

**Supplementary Table.** Details of PRAETORIAN score and BMI for 13 cases in the Change group

|  | Patients  (Age, years/Sex) | BMI, kg/m^2^ | Supine PS | Conventional PS | Change Step  (Primary Contributor) |
| --- | --- | --- | --- | --- | --- |
| 1 | 67 / male | 24.1 | 30 | 60 | Step1 |
| 2 | 62 / male | 25.9 | 30 | 60 | Step1 |
| 3 | 59 / male | 26.0 | 30 | 60 | Step1 |
| 4 | 44 / male | 31.1 | 30 | 60 | Step1 |
| 5 | 55 / male | 35.4 | 30 | 90 | Step1 |
| 6 | 43 / male | 40.8 | 30 | 90 | Step1 |
| 7 | 64 / male | 27.3 | 30 | 60 | Step2 |
| 8 | 56 / male | 31.1 | 30 | 60 | Step2 |
| 9 | 75 / male | 24.0 | 30 | 45 | Step3 |
| 10 | 62 / male | 25.6 | 30 | 45 | Step3 |
| 11 | 51 / male | 29.4 | 30 | 45 | Step3 |
| 12 | 73 / male | 30.0 | 45 | 30 | Step3 |
| 13 | 44 / male | 31.9 | 135 | 90 | Step3 |

The two cases in which the risk classification changed are highlighted in gray; both patients had markedly elevated BMI (>35 kg/m²).

In another two patients (Cases 12 and 13), the supine PRAETORIAN score was higher than the conventional score, reflecting changes at Step 3.

**Supplementary Figure.** Representative radiographs of patients with risk reclassification between supine and conventional PRAETORIAN score assessments.

Radiographs from patients #5 and #6 are shown. In both cases, standing radiography demonstrated an apparent increase in adipose tissue beneath the shock coil compared with supine radiography, resulting in a higher Step 1 score and reclassification from low risk on supine assessment to intermediate risk on conventional standing assessment.
